# Enhancing awareness of risk factors for polycystic ovarian syndrome: The critical need for attention among adolescents and parents in coastal Karnataka, India

**DOI:** 10.1177/22799036251401944

**Published:** 2025-12-14

**Authors:** Lisa Aslin Mascarenhas, Ananya Ann Mathew, Anju Saji, Reenu Baby, Jeslyn Joseph, Aju Thomas Abraham, Ansuya, Rakesh Datta

**Affiliations:** 1 Manipal College of Nursing, Manipal Academy of Higher Education, Manipal, Karnataka, India

**Keywords:** polycystic ovarian syndrome, adolescents and young women, risk factors, knowledge, female healthcare professionals

## Abstract

**Background:**

Despite its high prevalence (6% and 26%), PCOS is undiagnosed and thus takes longer to identify.

**Objective:**

To assess knowledge about the risk factors of polycystic ovary syndrome among healthcare professional students and to identify the association between demographic data and the knowledge scores concerning the risk factors for polycystic ovarian syndrome.

**Design and methods:**

A cross-sectional survey was conducted among 400 female healthcare professional students from Udupi Taluk, South India. The participants were recruited through a simple random technique. The baseline data and knowledge of risk factors for PCOS were measured using a validated and retested structured questionnaire. The data were analyzed using descriptive and inferential statistics using SPSS version 16.

**Results:**

The mean age of the study participants was 20.31 ± 1.37 years. The study demonstrated that 18.8% were overweight, 11.3% were suffering from PCOS, and 11.3% had a family history of PCOS. Approximately 75.5% of the participants had an average knowledge of PCOS, and 2.3% had poor knowledge. Sixty-six percent of the participants were not aware that dietary practices influence PCOS, various risk factors (66%), preventive measures (83.5%), risks associated with pregnancy due to PCOS (81.2%), measures to control the complications of PCOS (66.2%), complications of PCOS (57.5%), and the type of food used as a risk factor for PCOS (55%).

**Conclusions:**

Adolescents and young women are not very aware of various risk factors, complications, and measures to control the complications of PCOS. Periodic screening and successful awareness programs will facilitate improved understanding, increased diagnosis, and effective management of PCOS.

## Introduction

Polycystic ovary syndrome (PCOS) is a serious public health concern and one of the most prevalent hormonal disorders affecting women of reproductive age. The prevalence of PCOS in Indian adolescents is 9.13%, which is relatively common. The global prevalence of PCOS is estimated to be between 6% and 26%.^1^ Polycystic ovary syndrome affects all races and cultural groups. As calculated, 6%–12% or 5 million people in the USA suffer from PCOS.^2^ In India, 22.5% of people suffer from PCOS. The National Institute of Health (NIH) estimated that 4%–20% of women of reproductive age are affected by PCOS.^3^ A previous study in Udupi, Karnataka, reported that 13.6% of pre-university college students were at moderate risk for developing PCOS.^4^ A systematic review and meta-analysis reported that the global prevalence of PCOS ranges from 6% to 10%. The severity of this problem may be even greater.^5^

PCOS has challenging physical symptoms, including hirsutism, hair loss, acne, weight gain, and irregular menstruation, all of which are triggered by elevated androgen hormones. In the future, one of the causes of infertility will be PCOS. There are also many complications, such as type 2 diabetes, hypertension, and endometrial cancer.^6^ The diagnosis of PCOS depends on a combination of clinical symptoms, biochemical markers, and imaging (Rotterdam criteria), such as androgen elevation, anti-Mullerian hormone elevation, and elevated stromal-to-surface area on ultrasound.^7–12^ Young adolescents diagnosed with PCOS display numerous risk factors, such as insulin resistance, dyslipidemia, type 2 diabetes mellitus, and obesity, which are critical for early identification and treatment to avoid cardiovascular complications in adulthood.^13–15^ Early identification and treatment, comprising family history and risk assessments, and laboratory tests, are crucial for handling PCOS in girls.^16–18^ This comprehensive approach can aid in timely diagnosis and intervention, possibly preventing severe cardiovascular and metabolic health problems.^16–18^

Despite its high prevalence, PCOS is underdiagnosed and thus takes longer to identify. The prevalence of PCOS is similar to an iceberg phenomenon in the community^19^ Diagnosing PCOS is challenging, as PCOS cannot be diagnosed with a single diagnostic indicator due to the overlay of PCOS signs and symptoms with typical pubertal variations, and a lack of awareness of PCOS and its complications among youths.^19–23^Adolescent girls or their mothers are more likely to misdiagnose this problem as other health problems.^19^ This tends to result in underdiagnosis or overdiagnosis, causing frustration and delays in treatment^18–23^ This draws attention to the issue of early diagnosis in adolescent girls.^1^

Young female students/girls must handle academic tasks and numerous other demands during adolescence and adulthood. They might also be unaware of alarming symptoms, have an inferiority complex about their physical appearance, suffer from some problems, feel isolated by other issues, or be uncertain about treatment. Owing to these factors, young women may have difficult and stressful times.^24^ Academic stress and POCD symptoms contribute to the deterioration of a student’s quality of life.^25^

The incidence of PCOS is increasing in the Indian population. Treatment for PCOS is complex and often personalized to a person’s symptoms and goals, such as managing infertility or reducing androgen levels. It can be managed with diet, lifestyle changes, exercise, and medications such as antiandrogens and oral contraceptives with or without low-dose metformin.^26–28^ Delays in diagnosis can lead to the progression of the condition, that is, PCOS and comorbidities, making it more difficult to implement lifestyle interventions, which are critical for improving PCOS features and quality of life. The disorder and its associated comorbidities increase healthcare costs and reduce quality of life. It is good if all reproductive-aged women are aware of problems related to the reproductive system, such as PCOS, and their risk factors, so that they can manage their health themselves by making diet modifications, exercise, and treatment by consulting an obstetrician.^11^ With this background, researchers intend to investigate the knowledge about risk factors of PCOS so that further measures can be taken to improve them; improving the population’s overall health contributes to attaining Sustainable Development Goal 3.

## Primary objective

To assess the knowledge about the risk factors of polycystic ovary syndrome among healthcare professional students

## Secondary objective

To identify the association between the demographic data and knowledge scores concerning the risk factors of polycystic ovary syndrome.

## Materials and methods

A cross-sectional survey was conducted between December 2023 and 31st January 2024 among female healthcare professional students.

## Study participants and sampling

The study population consisted of female undergraduate students at healthcare institutions. Initially, using a simple random sampling technique and the lottery method, four colleges were selected from the list of health science colleges. Then, the courses are taken as strata. Finally, students were recruited using simple random sampling. The sample size was calculated using Cochran’s formula.

$$n=(z^{2}\times p\times (1-p))/d^{2}$$

*z*: 95% confidence interval, *p̂*: sample proportion (48%), *d*: margin of 5% and 15% attrition, *N* = 384, and 400 samples were included in the study.

In the first step, 4 health care teaching institutions were selected from 13 institutions of the Manipal Academy of Higher Education using a simple random technique and a lottery method. The list of courses was collected from the head of the institution. In the next step, stratified proportionate sampling was employed. Undergraduate courses were considered strata. Courses were selected randomly from the institutions. After obtaining the students list from the undergraduate program coordinator, subjects from each stratum were taken proportionately from each batch, that is, BSc Nursing (100), MBBS(100), Diploma in Pharmacy (107), and Allied Health Science (93). Furthermore, the subjects from each course were selected using a simple random sampling method by computer-aided random selection. A total of 400 female students participated in this study. Students who did not consent were excluded from this study.

Inclusion criteria:

- Undergraduate students 18–30 years.
- Those who are willing to participate in the study.
- Female healthcare professionals’ students studying in the Manipal Academy of Higher Education.

Exclusion criteria: Participants who are not willing to participate in the study.

All methods were carried out in accordance with relevant guidelines and regulations. This study was designed and reported in accordance with the STROBE guidelines (https://www.equator-network.org/reporting-guidelines/strobe/).

## Data collection procedure

**Data collection instruments:** The researchers self-developed a demographic proforma and structured knowledge questionnaire to collect data. In section A of the demographic proforma, information on age, course, place of residence, weight, and height was collected, and information on suffering from PCOS, age of attained menarche, regularity of menstruation, and history of PCOS in the family was recorded in section B of the demographic proforma. The knowledge of risk factors and the management of PCOS were measured using a de novo developed structured knowledge questionnaire. Tools were given to seven experts to obtain their suggestions and opinions. Content validity was computed on the basis of the agreement and disagreement given by the experts. All the items were agreed upon by the experts except for minor modifications/corrections. The content validity index(S-CVI) of the knowledge questionnaire’s scale was 1. Reliability was calculated using the split-half method using the Spearman-Brown prophecy formula, and the estimated *r* value was 0.92. Data were collected after participants agreed, and written informed consent was obtained from all participants prior to data collection, with assurances of privacy and the strict maintenance of confidentiality throughout the study.

Weight and height were recorded using a standard calibrated weighing scale and a stadiometer. Information on sociodemographic and knowledge of risk factors and management was collected by administering the tools. BMI was categorized according to the WHO classification. The knowledge was interpreted as good (≥66% of the score ), average (≥33%–65%), and poor (<33%).

## Ethical considerations

Administrative permission from the study setting was obtained. The Institutional Research Committee (IRC 374/2022) and Institutional Ethics Committee (IEC2 - 177) approved this study. The study was registered with the Clinical Trials Registry India (CTRI). Informed consent was obtained from all the participants involved in the study, assuring and maintaining privacy and confidentiality. The participants were clearly informed about the purpose of the study, their rights, and how their data would be used. No identifying information (such as name, address, or phone number) was recorded in the dataset. Participation was solely voluntary, and written consent was obtained before data collection.

**Data analysis** SPSS version 16 is widely available and often provided under institutional licenses, making it accessible for students and researchers. The data were entered and analyzed using SPSS version 16.0. The study used descriptive statistics such as frequencies and percentages to describe the sample characteristics. A test of the associations between various determinants and PCOS among students was computed using the chi-square test. *p* ≤ 0.05 was taken as the cutoff for a significant association. The mean, standard deviation, and class interval were used to estimate the precision and reliability of the sample in terms of item-wise knowledge of PCOS.

## Results

### Frequency and percentage of study sample characteristics

The mean age of the study participants was 20.31 ± 1.37 years. Among the 400 participants, 18.8% were overweight. The majority of participants attained menarche between the ages of 11 and 15 years. A few participants, 8 (2%), attained menarche at 10 and 9 years, and 2.75% reached menarche at 16 years. It was observed that 7.8% were experiencing irregular menstrual cycle. The present study revealed that 11.3% of the participants had PCOS, and 11.3% had a family history of PCOS (Table 1).

**Table 1.** Frequencies and percentages of study sample characteristics *N* = 400.

Sample characteristics	Frequency	Percentage
Age in years	Mean ± SD	20.31 ± 1.37
Course studying		
BSc Nursing	100	25.0
Allied Health Science	93	23.3
MBBS	100	25.0
Diploma in Pharmacy	107	26.7
Place of residence		
Urban	326	81.5
Rural	74	18.5
BMI		
Underweight	73	18.3
Normal	252	63.0
Overweight	75	18.7
Menarche age (in years)		
9–10	8	2.0
11–15	381	95.25
16	11	2.75
Frequency of menstruation		
Every month	369	92.2
Once in 15 days	3	0.8
2–3 months	22	5.5
More than 3 months	6	1.5
Students suffering from PCOS		
Yes	45	11.3
No	355	88.7
Family history of PCOS		
Yes	45	11.3
No	355	88.7
Family member having PCOS *n* = 45		
Aunt	11	
Grandmother	01	
Mother	10	
Sister	23	

### Levels of knowledge about risk factors for PCOS among healthcare professional students

**Figure 1.**
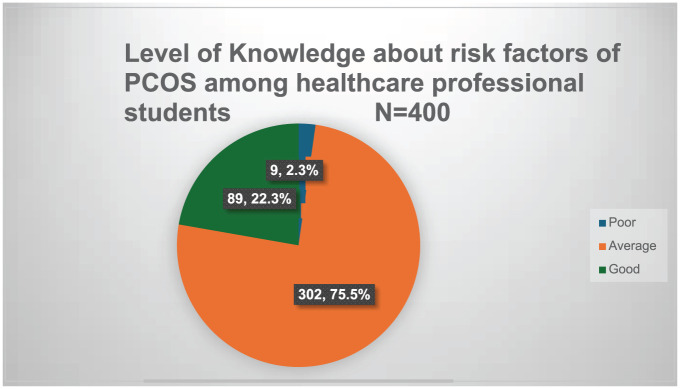
Level of knowledge about risk factors for PCOS among healthcare professional students *N* = 400.

The level of knowledge score was categorized as poor, good, and average, with a mean and SD of 19.86 ± 3.53 depicted in Figure 1. The minimum possible score is 6, and the maximum possible score is 32.

### Area-wise distribution of knowledge scores on risk factors for PCOS

Table 2 displays the results of the study, item-wise analysis of knowledge of risk factors and management. The participants responded correctly to the causes for PCOS 94.8% (95% CI: 0.93–0.97, *p* = 0.00), the major predisposing factor 81.8% (95% CI: 0.78–0.86, *p* = 0.00), the cause for PCOS in reproductive women 72.8% (0.68–0.77, *p* = 0.00), and the problem/complication likely to occur in people with PCOS 92.8% (95% CI: 0.9–0.95, *p* = 0.00). Most of the participants (88.2%) were aware of the common methods used for diagnosis, various lifestyle management (88.2%), and symptoms of PCOS (82.2%).

**Table 2.** Area-wise distribution of knowledge scores on risk factors for PCOS *N* = 400.

Knowledge areas	Responses				
	Correct *f* (%)	Incorrect *f* (%)	Mean ± SD	95% CI	*p* value
Causes for PCOS	379 (94.8)	21 (5.2)	0.92 ± 0.22	0.93–0.97	0.00
Food as a cause of PCOS	262 (65.5)	138 (34.5)	0.65 ± 0.47	0.61–0.70	0.00
Predisposing factors for PCOS	327 (81.8)	73 (18.2)	0.82 ± 0.38	0.78–0.86	0.00
Various risk factors for PCOS	136 (34)	264 (66)	0.34 ± 47	0.29–0.39	0.00
Cause for PCOS in reproductive women	291 (72.8)	109 (27.2)	0.73 ± 0.44	0.68–0.77	0.00
The problem can occur in people with PCOS	371 (92.8)	29 (7.2)	0.92 ± 0.26	0.90–0.95	0.00
The type of food might cause PCOS	180 (45)	220 (55)	0.45 ± 0.49	0.40–0.50	0.00
The common method to diagnose	353 (88.2)	47 (11.8)	0.88 ± 0.32	0.85–0.91	0.00
Lifestyle management	353 (88.2)	47 (11.8)	0.88 ± 0.32	0.85–0.91	0.00
Symptoms of PCOS	329 (82.2)	71 (17.8)	0.82 ± 0.38	0.78–0.86	0.00
Predominant clinical features	274 (68.5)	126 (31.5)	0.69 ± 0.46	0.64–0.73	0.00
Preventive measures	66 (16.5)	334 (83.5)	0.16 ± 0.37	0.50–0.60	0.00
The risk associated with pregnancy	155 (38.8)	245 (81.2)	0.39 ± 0.48	0.24–0.34	0.00
Complications of PCOS	170 (42.5)	230 (57.5)	0.42 ± 0.49	0.38–0.47	0.00
Measures to control the complications	135 (33.8)	265 (66.2)	0.34 ± 0.47	0.29–0.38	0.00
Drug of choice (medications) to be taken	223 (55.8)	177 (44.2)	0.56 ± 0.49	0.51–0.61	0.00
Lifestyle modification to be practiced	327 (81.8)	73 (18.2)	0.82 ± 0.38	0.78–0.86	0.00
Treatment in a severe case	210 (52.5)	190 (47.5)	0.53 ± 0.50	0.48–0.57	0.00

Furthermore, in many areas, the participants did not have good knowledge, which led to incorrect answers such as nutrient/food influences PCOS 65.5% (95% CI: 0.61–0.70, *p* = 0.00), various risk factors 66% (95% CI: 0.29–0.39, *p* = 0.00), preventive measures 83.5% (95% CI: 0.50–0.6, *p* = 0.00), risks associated with pregnancy due to PCOS 81.2% (95% CI: 0.24–0.34, *p* = 0.00), measures to control the complications associated with PCOS 66.2% (95% CI: 0.29–0.38, *p* = 0.00), complications of PCOS 57.5% (95% CI: 0.38–0.47, *p* = 0.00), and the type of food used as a risk factor for PCOS 55% (95% CI: 0.40–0.50, *p* = 0.00).

### Association between PCOS and BMI, age of menarche, family history of PCOS

The chi-square test was computed to determine the association between PCOS and BMI, menarche age, and family history of PCOS, as presented in Table 3. The findings revealed a significant association between PCOS and BMI (χ^2^_**(df)**_ = 10.51_(2)_, *p* = 0.005) and a family history of PCOS (χ^2^_**(df)**_ = 29.32_(2)_, *p* ≤ 0.000) irregular menstruation (χ^2^_**(df)**_ = 91.5_(1)_, *p* < 0.00). The association between BMI and family history of PCOS was significant at the 0.04 level (χ^2^_**(df)**_ = 4.62, *p* = 0.04).

**Table 3.** Association between PCOS and BMI, age of menarche, family history of PCOS among healthcare professional students with and without previously diagnosed polycystic ovary syndrome *N* = 400.

Variables	Self-reported previously diagnosed PCOS		χ^2^	Odds ratio (95% CI)	*p*-value
	Yes, *f* (%)	No, *f* (%)			
BMI status					
Underweight	04 (1)	69 (17.25)	10.51	0.71 (0.38–1.33)	0.005
Normal	25 (6.25)	227 (56.75)			
Overweight	16 (4)	59 (14.75)			
Menarche age					
<10 years	0 (0)	8 (2.0)	4.65	-	0.98
11–15 years	45 (11.2)	336 (84.0)			
>15 years	0 (0)	11 (2.8)			
Family history of PCOS					
Yes	14 (3.5)	31 (7.8)	29.32_(1)_	0.21 (0.10–0.44)	<0.001
No	31 (7.8)	324 (81.0)			
Menstruation irregularity					
Yes	20 (62.5)	12 (37.5)	91.5_(1)_	22.9 (10.0–52.1)	<0.001
No	25 (6.8)	343 (93.2)			
Normal	33 (6.25)	219 (56.75)			
Overweight	9 (4)	66 (14.75)			

A logistic regression with a 95% confidence interval was then carried out to adjust for confounders and identify the factors that were truly associated with PCOS. It is found that abnormal BMI (OR 0.71, 95% CI (0.38–1.33) *p* = 0.005), Family history of PCOS (OR 0.21, 95% CI (0.10–0.44) *p* < 0.001), and irregular menstruation (OR 22.9, 95% CI (10.0–52.1) *p* < 0.001). The findings revealed that having a mother, sister, or other close relative with PCOS significantly increases the risk. An abnormal BMI (obesity) may be due to a lack of physical activity or an unhealthy diet. Irregular menstruation also contributes to the risk of PCOS.

## Discussion

In our study, 11.3% of female healthcare professional students were suffering from and diagnosed with PCOS. This is supported by various research studies and reports as follows. In 2023, the World Health Organization reported that, polycystic ovary diseases affect an estimated 8%–13% of reproductive-aged women.^19^ In India, it is estimated that 3.7%–22.5% of women of reproductive age are affected by PCOS.^29^ An approximate 43% prevalence of polycystic ovarian morphology (PCOM) among healthy females was reported in a previous study.^7^ A study conducted in Malaysia also supported this finding, reporting that 10.49% of respondents had a medical diagnosis of PCOS, and that 2.68% were diagnosed with PCOS during data collection, while 32.93% were suspected of having PCOS.^30^ A study conducted in Tamil Nadu and Hyderabad, Bangalore, has a supportive finding on the prevalence of PCOS with 18%,11.5%, and 10.97% respectively, among young adolescent females.^1,31,32^ A study among medical students at a private medical college in South Karnataka reported that 8.1% were already diagnosed with PCOS and that 9.1% were at high risk.^33^ Another similar study from Udupi, Karnataka, reported that 13.6% of pre-university students had a moderate risk for PCOS.^4^ Many cases in the community remain undiagnosed; hence, PCOS/PCOS has become a common and undiagnosed disorder among young women, and this warrants periodic screening activities. Thus, risk assessment in the form of a survey would be the ideal strategy to identify this condition early and thus encourage young women to seek timely treatment and prevent its long-term complications.

Our study observed that a few risk factors are associated with its development. A total of 18.8% of the students were overweight, and 8% experienced irregular menstruation either once every 15 days or more than 2 months. A total of 45 participants reported a family history of PCOS. A significant association between PCOS and BMI and between a family history of PCOS and irregular menstruation was found in our study. Although the causes of PCOS are unclear, many studies have reported the links between PCOS and genetics, family history, and obesity.^34^ Students who were confirmed to have PCOS had BMIs that were considerably higher in both the Mangalore study by Joseph et al.^33^ and the Korean study.^35^ However, a study done in Trivandrum, India, reported no association.^36^ According to earlier research, overweight is the factor that affects the quality of life of young women with PCOS. People with PCOS have stated that they find this concern to be much more troubling than other disorders, such as irregular menstruation.^37^ Thus, it is evident that obesity, particularly abdominal obesity, is closely linked to PCOS and can worsen symptoms and metabolic complications. Having a family member, that is, a mother, sister, or other close relative, with PCOS significantly increases the risk for developing PCOS among adolescents and young women.

Awareness of PCOS risk factors, symptoms, and complications among students or young women is essential for early treatment and prevention of serious complications.^38^ In our study, 75.5% of female healthcare professional students had average knowledge, 2.3% had poor knowledge, and only 22.3% had good knowledge of the risk factors for PCOS and management. Although students are healthcare professionals, the majority have average knowledge levels; hence, it can be assumed that the knowledge of PCOS among young women may be poor. A study from Mangalore, India, reported that 76% of students had average knowledge and that 10.7% had good knowledge.^39^ Few studies have demonstrated low awareness of PCOS among adolescents and young women. A study of female adolescents in an educational institution reported that 31.2% had low knowledge about PCOS, and that 47.3% young women had poor knowledge in Malaysia.

This is also supported by a study conducted by Rakumari et al. from Odisha, who reported that 78% of school-going girls had never heard about polycystic ovary syndromes,^40^ 56% of Pakistani women did not know about PCOS,^41^ 28% of medical students were unaware of PCOS (21 medical students), 36% of the population had no idea about polycystic ovarian syndrome, and 58% had heard the name before and have minimal knowledge of the condition by Dhaka University students reported by Jahangir.^42^ Another similar study among medical students in Chennai by Bangaru et al. demonstrated that 57.8% of the participants had good knowledge of PCOS, whereas 36.9% had fair knowledge and 5.2% had poor knowledge about PCOS.^43^ A study conducted among students in Telangana reported that adolescents’ awareness was critically poor.^44^ Many studies have reported inadequate knowledge of PCOS among adolescents and young women, either medical or nonmedical students. This may be the reason for not maintaining a healthy lifestyle, including a healthy diet and exercise to reduce weight, and consulting an obstetrician for problems.

Item-wise analysis was done to determine more precisely the domain of knowledge of PCOS that the study participants lacked. Students were not very aware that junk food eating practices might cause PCOS. They do not have good knowledge of various risk factors for PCOS, preventive measures, the risks associated with pregnancy, and the different complications that can develop. Half of the students did not know about medication therapy or the drug of choice for treating PCOS. Jabeen et al. reported that 78.4% of adolescents and young girls attending schools and colleges in Telangana were unaware of PCOS, and they were unaware of the symptoms of PCOS, and the association of PCOS with cardiovascular complications, diabetes, and gynecological cancer. However, most are aware of healthy diets and physical activities and their ability to prevent and manage PCOS.^44^ Kiran et al. reported that knowledge of PCOS was very poor among Pakistani women.^45^ A study from Uttar Pradesh reported that study participants are less aware of PCOS, and 19.3% of participants had no idea about what PCOS is and had never heard about this condition. They found an association between knowledge of the manifestation of disorders and lifestyle variation.^46^

Importantly, people suffering from PCOS are fully aware of their condition, and they require support from colleagues, parents, etc., to improve their quality of life and succeed in their academics without distress during their studies. The support should be extended to weight management, healthy lifestyles, quality of life, and medication therapy.

## Limitations

The study was limited to health care professional students and employed a cross-sectional design.

The study gathered **self-reported previously diagnosed** polycystic ovary syndrome and did not involve the diagnosis of the case.

## Implications and recommendations

It is possible to integrate in Ayush Bharath insurance and be involved in the school screening program to detect and treat PCOS in the early stage. Reproductive health and PCOS awareness should be incorporated into health education modules for high school and college students.

## Conclusions

This study demonstrated that PCOS is a common disease and that knowledge of PCOS is inadequate in adolescents and young women. Integrating PCOS awareness into health education programs is essential to address the growing burden of reproductive and metabolic health issues among adolescents. Therefore, periodic screening is needed, particularly for those with a family history and a high BMI. Health education also reduces stigma and misconceptions, promoting an open dialog on women’s health. By incorporating PCOS awareness into school, college, and community-based programs, institutions can empower young women with knowledge about healthy lifestyle practices, early screening, and the importance of regular medical checkups. This approach not only enhances individual well-being but also contributes to reducing the long-term risk of infertility, diabetes, and cardiovascular complications associated with PCOS.
